# Abdominal angina in occlusive mesenteric vascular disease: a case report

**DOI:** 10.1186/1757-1626-2-82

**Published:** 2009-01-23

**Authors:** Bjoern Kitzing

**Affiliations:** 1 Westmead Hospital, Cnr Hawkesbury and Darcy Roads, Sydney, New South Wales, Australia

## Abstract

**Introduction:**

Abdominal angina is a descriptive term for abdominal pain that can occur postprandially in patients with occlusive mesenteric vascular disease due to insufficient increase in blood flow.

**Case presentation:**

In this case a 60-year-old Caucasian woman with a 2 year history of abdominal angina presented to hospital for elective mesenteric revascularization surgery. Postoperative recovery was complicated by graft occlusion resulting in hepatic ischemia as well as splenic and small bowel infarction.

**Conclusion:**

This case highlights the importance of keeping this differential diagnosis in mind when dealing with patients who have a long history of abdominal pain and discusses some of the complications that may occur after surgical treatment.

## Introduction

This is a rare case of a patient with abdominal angina secondary to occlusive mesenteric vascular disease. The diagnosis was made after a 2 year history of abdominal pain. Elective revascularization surgery was performed and the complications that occurred are discussed.

## Case presentation

A 59-year-old Caucasian woman with a history of intermittent abdominal pain and significant weight loss for the last two years was admitted to hospital for elective aorto-mesenteric bypass surgery. Over the course of the two years she had presented to multiple doctors who had performed various procedures including gastroscopy and cholecystectomy without finding and eliminating the course for the patient's symptoms. A CT scan was performed which showed focal narrowing of the coeliac trunk. The patient was then referred to our hospital for a mesenteric artery angiogram. This demonstrated a high grade stenosis of the proximal coeliac artery, complete occlusion of the superior mesenteric artery and hypertrophy of the inferior mesenteric artery with evidence of a wondering artery of Drummond supplying the branches of the superior mesenteric artery and thus confirmed the suspected diagnosis of occlusive mesenteric vascular disease.

The patient described the abdominal pain as intermittent, mostly appearing 20 to 30 minutes after eating big meals. As a consequence she had begun to associate food with pain and had developed sitophobia (fear of food) resulting in significant weight loss. Her medical history was also significant for smoking and hypercholesterolaemia.

On physical examination in pre-admission clinic the patient appeared cachectic. Her blood pressure was 110/80 on the right side and 105/75 on the left, pulse was 80 beats/min and regular, respiratory rate was 15 breaths/min and oral temperature was 36.7°C. Her examination also revealed normal jugular venous pressure and normal breath sounds over both lung bases. Heart sounds were distant, and peripheral pulses were normal. The remainder of the examination findings were unremarkable.

The ECG performed showed normal sinus rhythm without any ST-segment changes. Blood results including liver function tests and coagulation studies were normal. The chest radiograph obtained showed no abnormalities.

After routine preparations for surgery the patient went to theatre and mesenteric revascularization was performed with an antegrade prosthetic graft bypass. The proximal trunk of the small-calibre bifurcated prosthetic graft was anastomosed to the supracoeliac aorta, and the distal limbs were sewn to the coeliac artery and superior mesenteric artery, just beyond the stenotic segments. Intraoperative duplex ultrasound examination confirmed the technical adequacy of the revascularization. Postoperatively the patient was transfered to the intensive care unit. However, the next day blood tests showed a steep rise in her liver enzymes (ALT 1792 U/L, AST 2731 U/L) suggestive of hepatic ischemia and duplex ultrasonography confirmed the suspected diagnosis of graft occlusion. The patient was taken back to theatre for emergency thrombendarterectomy. Both limbs of the graft were found to be occluded and the coeliac limb was shortened. Overnight the arterial blood lactate level began to increase so that a laparotomy was performed revealing a dusky small bowel segment which was resected. The patient was commenced on transparenteral nutrition and remained stable until 18 days post initial surgery when signs of sepsis and peritonitis became evident 1. Laparotomy was again performed resulting in splenectomy, resection of the small bowel anastomosis and abdominal wash-out. The recovery period was then prolonged but without further complications and the patient was transferred for rehabilitation.

**Figure 1:**
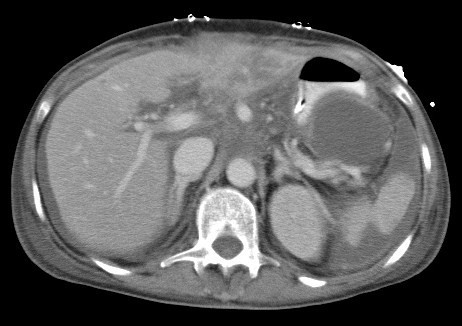
**Select axial image of computed tomography showing ischemic necrosis of the left lobe of the liver and infarction of the splenic parenchyma post prosthetic graft occlusion**.

## Conclusion

Abdominal angina is a term that describes postprandial pain in patients with occlusive mesenteric vascular disease and is generally attributed to Baccelli (1918). Surgical treatment of this condition was first proposed in 1957 by Mikkelsen and the first thrombendarterectomy of the superior mesenteric artery was reported in 1958. The abdominal pain is a consequence of blood flow not being able to meet visceral demand and is therefore thought to be similar to angina pectoris in patients with coronary artery disease or claudication in peripheral vascular diesease. Atherosclerotic vascular disease is the most common cause and as such smoking is an associated risk factor with 75–80% of affected patients admitting to smoking.

The three main arteries supplying the gut are the coeliac, superior mesenteric and inferior mesenteric. Collateral circulation between the coeliac and superior mesenteric arteries as well as between the superior and inferior mesenteric arteries generally ensures adequate blood flow to the gut, unless significant stenoses or occlusions of 2 of the 3 vessels are present.

Many patients have to wait between 1.5 and 2 years before the diagnosis is made because they are initially thought to have a malignancy. The presenting symptoms of abdominal pain, weight loss and an age older than 60 years lead to investigations that are not diagnostic for occlusive mesenteric vascular disease. The criterion standard test is mesenteric artery angiogram.

Mesenteric revascularization relieves the pain and may prevent intestinal infarction. The role of percutaneous transluminal angioplasty (PTA) is currently under investigation and the follow-up data is limited [1]. The surgical treatment options include transarterial endarterectomy, retrograde bypass, antegrade bypass and trapdoor aortotomy [2-4]. Antegrade prosthetic graft bypass being the most common procedure performed. Postoperative complications include ileus, bleeding, coagulopathy, pulmonary insufficiency, renal and hepatic failure. In some cases graft occlusion may be due to competing arterial flow systems post revascularization.

Magnetic resonance imaging and duplex ultrasonography have been used for outpatient follow-up but are generally not recommended because the proper treatment of an asymptomatic occlusion post surgery is not known.

To conclude, this is a rare case of abdominal angina due to occlusive mesenteric vascular disease. The clinical pathway demonstrates the importance of keeping this differential diagnosis in mind when dealing with patients with a long history of abdominal pain and also highlights the risks involved in the surgical treatment of this condition.

## List of abbreviations

CT: computed tomography; ECG: electrocardiogram; ALT: alanine transaminase; AST: aspartate aminotransferase; PTA: percutaneous transluminal angioplasty.

## Competing interests

The author declares that they have no competing interests.

## Consent

Written informed consent was obtained from the patient for publication of this case report. A copy of the written consent is available for review by the Editor-in-Chief of this journal.

## Authors' contributions

BK made substantial contributions to conception and design, drafted the manuscript, revised it critically for important intellectual content and gave final approval of the version to be published.
